# 3D Plotting of Silica/Collagen Xerogel Granules in an Alginate Matrix for Tissue-Engineered Bone Implants

**DOI:** 10.3390/ma14040830

**Published:** 2021-02-09

**Authors:** Sina Rößler, Andreas Brückner, Iris Kruppke, Hans-Peter Wiesmann, Thomas Hanke, Benjamin Kruppke

**Affiliations:** 1 Max Bergmann Center of Biomaterials and Institute of Materials Science, Technische Universität Dresden, 01069 Dresden, Germany; Sina.Roessler@tu-dresden.de (S.R.); Andreas.Brueckner@tu-dresden.de (A.B.); Hans-Peter.Wiesmann@tu-dresden.de (H.-P.W.); Thomas.Hanke@tu-dresden.de (T.H.); 2 Institute of Textile Machinery and High Performance Materials Technology, TU Dresden, 01069 Dresden, Germany; Iris.Kruppke@tu-dresden.de

**Keywords:** silica/collagen composite, additive manufacturing, biomaterial ink, rheology, tissue-engineered bone implant

## Abstract

Today, materials designed for bone regeneration are requested to be degradable and resorbable, bioactive, porous, and osteoconductive, as well as to be an active player in the bone-remodeling process. Multiphasic silica/collagen Xerogels were shown, earlier, to meet these requirements. The aim of the present study was to use these excellent material properties of silica/collagen Xerogels and to process them by additive manufacturing, in this case 3D plotting, to generate implants matching patient specific shapes of fractures or lesions. The concept is to have Xerogel granules as active major components embedded, to a large proportion, in a matrix that binds the granules in the scaffold. By using viscoelastic alginate as matrix, pastes of Xerogel granules were processed via 3D plotting. Moreover, alginate concentration was shown to be the key to a high content of irregularly shaped Xerogel granules embedded in a minimum of matrix phase. Both the alginate matrix and Xerogel granules were also shown to influence viscoelastic behavior of the paste, as well as the dimensionally stability of the scaffolds. In conclusion, 3D plotting of Xerogel granules was successfully established by using viscoelastic properties of alginate as matrix phase.

## 1. Introduction

Bone is a natural organic/inorganic composite material that exhibits a complex hierarchical structure, ranging from arrangements in the nanometer range to structures on a macroscopic level. At the lowest level, polypeptide chains are wound together to form triple helices, which in turn are assembled into fibrils. The collagen fibrils act as templates for mineralization, in which the apatite crystals grow on the collagen fibrils. These mineralized collagen fibrils assemble into fibril bundles, which in turn form osteons. The osteons represent the basic structural unit of the cortex, also compact bone, and consist of concentric bone lamellae arranged around the central Haversian canal. The Haversian canals run through blood vessels and nerve cords. The cancellous bone has a spongy network of trabeculae, which consist of lamellar bone [1,2]. This highly porous trabeculae network with approximately 50–90% porosity [3] is filled with bone marrow, in which also hematopoietic and mesenchymal stem cells find their origin [4,5]. The complex hierarchical structure of bone is a reason for its special mechanical stability combining elasticity and strength [6]. The mechanical properties of bone originate from its structure. The outer compact bone exhibits elastic modulus of 3–30 GPa and the inner cancellous bone has elastic modulus of 0.02–2 GPa. In addition, the construction of cancellous bone enables the exchange of substances and the supply of the bone cells [7]. Bone tissue is subject to a constant remodeling process in which bone is continuously replaced by osteoclasts and is rebuilt by osteoblasts.

Silica/collagen composites produced by sol–gel process are biomimetic materials of organic and inorganic components, equipped with a structure over several structural levels [8]. So far, the focus has been on monolithic silica/collagen Xerogels, which have been investigated for their suitability as bone substitute materials [8,9,10,11,12]. Owing to their hybrid character and their structure over several structural levels, silica/collagen Xerogels have mechanical strengths in areas relevant to the bone [9]. Moreover, silica/collagen Xerogels have been shown to be bioactive, degradable, and resorbable, as well as to affect proliferation and osteogenic differentiation of human bone marrow stromal cells and osteoclastogenesis [9,10,11,12]. With all these properties, silica/collagen Xerogels match a lot of requirements to an ideal bone implant. However, macroporosity with pore sizes about 200–350 µm is another critical and desirable parameter for the success of bone implant. These structure sizes and resulting void spaces are mainly relevant for cell ingrowth and their nutrition especially in large biomaterial constructs. So far, this was lacking in case of monolithic silica/collagen Xerogels.

The fabrication of macroporous implants can be associated with scaffolds, which are the key elements of tissue engineering. For such tissue-engineered bone implants, various manufacturing methods have been applied in the last decades. However, methods like freeze drying, electrospinning, salt leaching, gas foaming or fiber deposition are not suitable to fabricate accurate scaffolds with a designed macroporosity and with defined pore sizes [13]. Moreover, properties related to the individual adjustment to diseases and/or patients gain importance [14]. To match patient specific shapes of fractures or lesions, as well as material macroporosity, interconnected pores, and defined pore sizes, additive manufacturing, especially 3D printing, is a widely used method [15].

By the help of additive manufacturing, material is added in a layer-by-layer manner to create a 3D implant [16]. Three-dimensional printing, a type of additive manufacturing, has the focus on printing by the help of inks. In this way, scaffolds can be fabricated by controlling shape, porosity, and pore size, as well as the interconnectivity of pores. One key element of 3D printing is the ink used. Therefore, a wide range of biomaterial inks were developed [17]. Biomaterial inks to create bone scaffolds combine both requirements on bone implant materials at all like excellent performance in mechanical stability, bio- and cytocompatibility, and degradation and resorption, as well as requirements on their processing. Requirements on processing are dependent on the type of 3D-printing technique.

To process biomaterial inks and to fabricate bone implants by 3D printing, currently, there are three main techniques: material jetting, material extrusion, and vat polymerization. Material jetting is a computer controlled technique based on the precise deposition of biomaterial ink droplets of microliter volumes onto a substrate [18]. Material jetting is widely applied in tissue engineering, using mostly hydrogel inks, like gelatin or alginate. Material extrusion is another computer controlled 3D-printing technique. Biomaterial inks for extrusion-based 3D printing are usually pastes, solutions, or dispersions that can be extruded by the coordinated motion of pneumatic pressure or plunger- or screw-based pressure through a nozzle or a needle onto a substrate [19]. The extruded biomaterial ink forms a continuous filament that is applied layer-by-layer to build 3D patterns that in turn form the scaffold in the desired shape. A fine resolution can be reached by the usage of related microscale nozzles or needles. The third technique—vat polymerization—is based on a liquid photopolymer resin stored in a vat. This liquid polymer is selectively polymerized at the surface of the vat. Polymerization is induced by a low-power ultraviolet (UV) light source [20]. A new thin layer is spread over the solid surface, while the *z*-axis is moving down. This process is repeated to fabricate a complete scaffold. The resolution of this technique varies from pico- to micro-scale, mostly depending on the used material [21].

In the present study, the advantage of excellent material properties of multiphasic silica/collagen Xerogels is to be combined with the advantages of extrusion-based 3D plotting. Thus, it is a successful first-time proof of principle to process granules made of silica/collagen composites in a viscoelastic matrix by 3D plotting.

## 2. Materials and Methods

### 2.1. Preparation of Xerogel/Alg Paste

**Figure 1:**
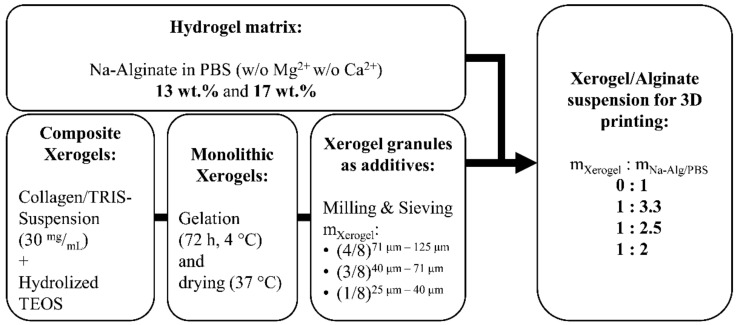
Scheme of hydrogel and Xerogel processing for preparation of 3D-plottable suspensions of various mass ratios of alginate to PBS and alginate hydrogel to Xerogel granules.

First, Xerogel monoliths were prepared as described previously and shown in Figure 1. In brief, bovine tropocollagen type I (GfN Herstellung von Naturextrakten GmbH, Wald-Michelbach, Germany) was dialyzed (MWCO 12–14 kDa, Roth, Karlsruhe, Germany) against deionized water for 7 days, fibrillized (30 mM neutral sodium phosphate buffer solution), and then lyophilized (until frozen liquid was removed; Christ Alpha1-4 laboratory freeze-dryer, Martin Christ Gefriertrocknungsanlagen GmbH, Osterode am Harz, Germany). Collagen was resuspended in 0.05 M Tris (pH 8, Roth, Karlsruhe, Germany) to a homogenous suspension (30 mg/mL). Silicic acid was prepared by hydrolysis of tetraethoxysilane (TEOS, 99%, Sigma-Aldrich, St. Luis, MO, USA; molar ratio TEOS/water = 1/4) under acidic conditions (0.01 M HCl). TEOS colloids were increasingly crushed by vigorous stirring (magnetic stirrer, 700 rpm), leading to a cloudy solution. After approximately 20 min, hydrolysis took place. Owing to the polymerization processes, there is always a mixture of silicic acids with different degrees of polymerization. To decrease polymerization processes, the solution was immediately cooled on ice for 30 min and then used for Xerogel production within one hour. For hydrogels with biphasic composition Xerogel, 30 wt.% bovine collagen and 70 wt.% silica were vigorously stirred, using a vortex mixer. After stabilization for 3 days, a climate chamber (Espec SH-221, Kita-ku, Japan) at 37 °C and 95% relative humidity, followed by a climate ramp, to achieve ambient conditions, was used to dry hydrogels until mass constancy. Afterwards, Xerogel granules were produced by grinding Xerogel monoliths, using a mixer mill (MM 400, Retsch, Haan, Germany). The resulting Xerogel granules were classified by means of a vibratory sieve shaker (AS 200, Retsch, Haan, Germany) and mesh sieves (25, 40, 71, and 125 μm).

Alginate (alginic acid sodium salt from brown algae, Sigma-Aldrich, St. Luis, MO, USA) was dissolved in phosphate buffered saline (PBS, Biochrom GmbH, Berlin, Germany), under vigorous stirring, to obtain a homogenous solution as matrix phase for the biomaterial ink. For degassing, alginate was centrifuged (900 rcf, 2 min). For biomaterial ink preparation, Xerogel granules were added to alginate in various mass ratios of Xerogel granules to alginate (Xerogel/Alg ratio) and distributed homogeneously, hereafter referred to as Xerogel/Alg paste. Xerogel/Alg paste was transferred to cartridges and centrifuged (1200 rcf, 3 min).

### 2.2. Rheological Characterization of Xerogel/Alg Pastes

A plate rheometer (Haake Mars II, Thermo Fisher Scientific, Waltham, MA, USA) with plate cone diameter of 20 mm and plate-plate-distance of 1 mm was used for rheological characterization of Xerogel/Alg pastes. Viscosity of pastes was measured in rotation mode with shear rates in ranges of 0.01–40 s^−1^. Amplitude sweep was used to evaluate viscoelastic region. Therefore, storage modulus as well as loss modulus were measured in oscillation mode for shear stress in ranges of 0.1–25,000 Pa working with constant oscillatory frequency of 1 s^−1^.

### 2.3. Scaffold Fabrication

For extrusion, Xerogel/Alg pastes were filled in 10 mL cartridges with conical needles with 410–840 µm in inner diameter (Globaco GmbH, Rödermark, Germany). Extrusion was performed via compressed air (70–540 kPa air pressure). Scaffolds (6 × 6 mm^2^) with alternating layer pattern (0°/90°, ABAB) were fabricated with a 3DDiscovery (RegenHU, Villaz-St-Pierre, Swiss), working with 0.5–4 mm/s print head speed. Plotting parameters are summarized in Table 1. After plotting, scaffolds were immersed in CaCl_2_ solution (1 M) for 5 min, to allow crosslinking, and dried at 37 °C (Xerogel/Alg scaffold). To estimate the dependency of the thickness of a plotted strand and the nozzle size, photographs of as-printed scaffolds, as well as of SEM images of dried scaffolds, were analyzed, using ImageJ (Vers. 1.53e, W. Rasband, National Institutes of Health, Bethesda, MD, USA). Strand diameter was measured at least 6 times each in horizontal and vertical orientation for calculating mean and standard deviation as a function of printing parameters.

### 2.4. Scanning Electron Microscopy

The 3D-plotted Xerogel/Alg scaffolds were characterized for surface morphology, as well as using a cross-section obtained by cutting with a diamond wire saw. Samples carbon coated on aluminum stubs were studied by using an ESEM XL 30 (Thermo Fisher Scientific, Waltham, MA, USA) scanning electron microscope (SEM). SEM investigations were carried out under high vacuum, an acceleration voltage of 3 kV, and detecting secondary electrons.

## 3. Results

### 3.1. Paste Preparation for 3D Plotting

Concentrations of alginate dissolved in PBS at 13 and 17 wt.% were best suited to achieve good handling. In the idle state, alginate of lower concentrations than 13 wt.% showed a minimum of dimensional stability, as well as a greater delay in flow behavior. Alginate concentrations higher than 17 wt.% showed dimensional stability in the idle state, but were not suitable for the extrusion through small conical dispensing needles. Needles with more than 840 µm in inner diameter have to be used, but, in turn, they will cause strands with increased diameters. In consequence, a decrease in pore sizes of the defined scaffold would occur, which was not intended to be plotted. The composition of Xerogel granules fractions of 50 wt.% of 125–71 µm, 37.5 wt.% of 71–40 µm, and 12.5 wt.% of 40–25 µm grain size were homogeneously dispersed in the continuous phase, namely alginate. Alginate and the grain distribution allowed the densest packing of Xerogel granules still suitable for extrusion-based plotting.

### 3.2. Pre-Plotting Properties

**Figure 2:**
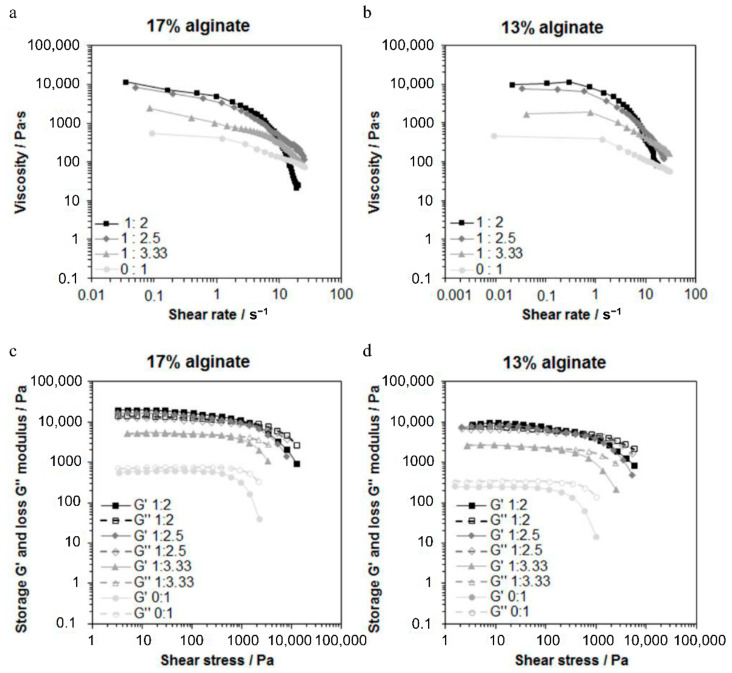
Rheological properties of Xerogel/Alg pastes. (**a**,**b**) Viscosities of Xerogel/Alg pastes with Xerogel/Alg ratios 1:3.33, 1:2.5, and 1:2, as well as of pure alginate (0:1) at 17 wt.% (**a**) and 13 wt.% (**b**) alginate concentration, respectively, for different shear rates. (**c**,**d**) Amplitude sweep measurements of Xerogel/Alg pastes with Xerogel/Alg ratios at 17 wt.% (**c**) and 13 wt.% (**d**) alginate concentration, respectively, for different shear stresses.

The viscoelastic properties of Xerogel/Alg pastes were characterized based on rheological measurements. Xerogel/Alg pastes of different component ratios, as well as of the different alginate concentrations, showed different viscosities (Figure 2a,b). With increasing concentrations of alginate dissolved in PBS increasing viscosities at low shear rate were observed. For Xerogel/Alg pastes of different component ratios, viscosity of the pastes increased with increasing amount of incorporated Xerogel granules. In general, Xerogel/Alg pastes made of 13 wt.% alginate showed Newtonian and shear-thinning behavior. For low shear rates, viscosity was independent from shear rate until zero shear viscosity and graphs exhibited Newtonian plateaus. With increasing shear rates, viscosity decreased. The linear decrease of viscosity with increasing shear rate in the logarithmic diagrams (Figure 2b) represents shear-thinning behavior. Xerogel/Alg pastes made of 17 wt.% alginate showed shear-thinning behavior only. For 13 wt.% alginate, zero shear viscosity increased with increasing Xerogel amount in the Xerogel/Alg paste, while for 17 wt.% alginate, no modification of Xerogel/Alg paste exhibited zero shear viscosity.

Amplitude sweep tests giving values of storage modulus G’ and loss modulus G’’ of shear modulus showed for different Xerogel/Alg pastes good equivalence with viscosities (Figure 2c,d). G’ > G’’ at low shear stress was observed for all Xerogel/Alg pastes made of 17 wt.% alginate (component ratios 1:3.33 to 1:2) as well as for Xerogel/Alg pastes made of 13 wt.% alginate with component ratios of 1:2.5 and 1:2. The opposite case (G’’ > G’ at low shear stress) was observed for pure 13 wt.% alginate and 13 wt.% alginate with component ratio of 1:3.33. For pure 17 wt.% alginate, G’ was very similar to G’’, with a slight dominance in G’’.

### 3.3. Plotting Properties

**Figure 3:**
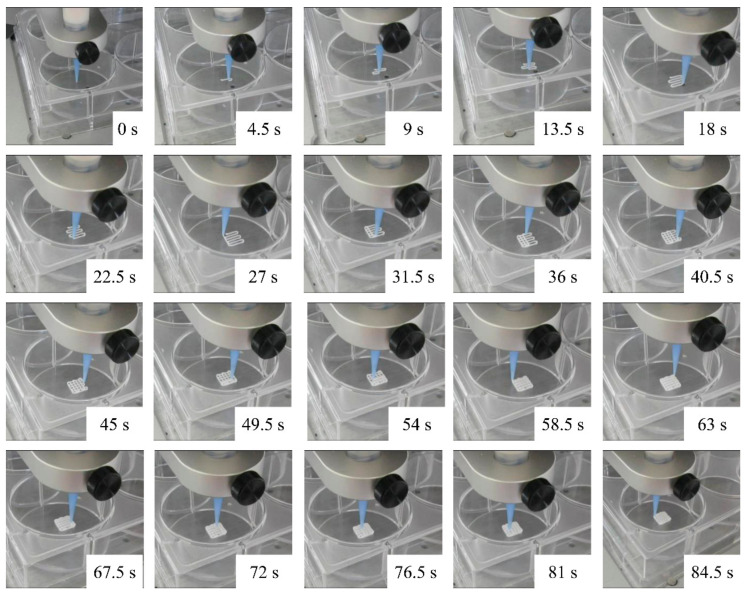
Time-lapse pictures of 3D-plotting process of a 6 mm x 6 mm, 6 strand scaffolds with an alternating layer pattern (0°/90°, ABAB).

Xerogel/Alg pastes were tested for their plotting suitability. Therefore, Xerogel/Alg pastes having component ratios of 1:3.33 to 1:2 were prepared, which could be uniformly extruded, using commercially available conical dispensing needles of sizes G22 (410 µm inner diameter) to G18 (840 µm inner diameter), to produce Xerogel/Alg scaffolds. A standard scaffold design with alternating layer pattern (0°/90°, ABAB) was selected as plottability test (Figure 3). The plottability test proved adhesion of the biomaterial inks on the polystyrene well plates and adhesion of strands to each other after finishing previous layers. Furthermore, for the plotting process, the layer height was adjusted in accordance to the needle diameter to produce individual layers, which do not penetrate into the layers below. This plotting process was assessed with the naked eye and evaluated with the experience gained previously. The printed scaffolds and the final dried scaffolds were additionally analyzed for their strand diameter, which was compared to the printing parameters (e.g., nozzle diameter).

In general, Xerogel/Alg pastes made of 17 wt.% alginate were limited in their suitability to generate 3D scaffolds. Pastes were hardly extrudable through conical dispensing needles of size G22 (410 µm inner diameter). In contrast, extrusion of Xerogel/Alg pastes made of 13 wt.% alginate was easy.

### 3.4. Post-Plotting Analysis

**Figure 4:**
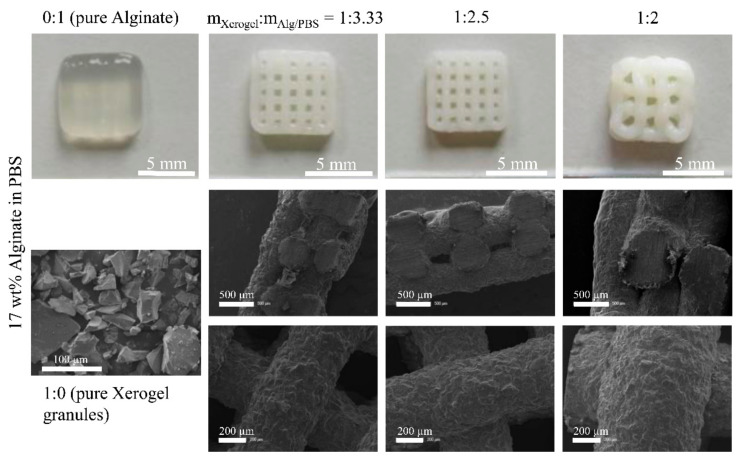
Photographs of 3D plotted biomaterial inks (upper row). Pure alginate (0:1) with 17 wt.% in PBS with little dimensional stability after 3D plotting and plotted composite scaffolds with Xerogel/Alg mass ratios of 1:3.33, 1:2.5, and 1:2. SEM images (lower rows) of pure Xerogel granules (<125 µm in size), as well as 3D plotted Xerogel/Alg scaffolds. Cross-section (middle row) and surface (lower row) of scaffolds containing a matrix of 17 wt.% alginate in PBS and Xerogel granules with component ratios of 1:3.33, 1:2.5, and 1:2.

**Figure 5:**
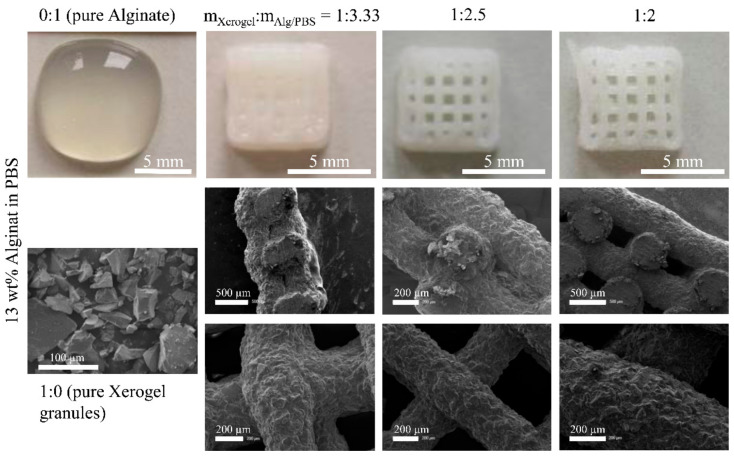
Photographs of 3D plotted biomaterial inks (upper row). Pure alginate (0:1) with 13 wt.% in PBS without dimensional stability after 3D plotting and plotted composite scaffolds with Xerogel/Alg mass ratios of 1:3.33, 1:2.5, and 1:2. SEM images (lower rows) of pure Xerogel granules (<125 µm in size), as well as 3D plotted Xerogel/Alg scaffolds. Cross-section (middle row) and surface (lower row) of scaffolds containing a matrix of 13 wt.% alginate in PBS and Xerogel granules with component ratios of 1:3.33, 1:2.5, and 1:2.

For Xerogel/Alg pastes made of 17 wt.%, alginate strands collapsed after extrusion (Figure 4). Although individual strands were visible on the surface, the strands sank into the layers below, leaving no lateral macropores (SEM images, cross-section of scaffolds; Figure 4 middle row). Pastes made of 13 wt.% alginate with Xerogel/Alg ratios of 1:2.5 and 1:2 were useful to generate 3D scaffolds with excellent dimensional stability (Figure 5). Xerogel/Alg 1:2 with 13 wt.% alginate showed in SEM images to be best suitable for 3D plotting. Cross-sections of scaffolds showed lateral macropores resulting from regularly extruded strands (Figure 5). Moreover, strands exhibited a coarsely structured surface formed by Xerogel granules (as visible in pure Xerogel particles SEM images in Figure 4 and Figure 5) homogenously distributed at a high content in the embedding alginate matrix. Strand diameter could not be measured in the case of pure alginate of 13 and 17 wt.% (Table 2), even though comparable biomaterial inks have been shown to form stable scaffolds [22]. Nevertheless, in almost all cases (except for 13 wt.% alginate with Xerogel/Alg ratios of 1:2, which had to be printed with increased needle diameter), after strand extrusion, an increase of the strand in comparison to the needle diameter of about 25–55% was observed. This relaxation of the biomaterial ink reduced the void phase of the scaffold. During drying, all scaffolds showed strand shrinkage of about 7–35%.

## 4. Discussion

The aim of the present study was to evaluate 3D plotting parameters to process silica/collagen composites. These silica/collagen composites were shown earlier to be bioactive, degradable, and resorbable, and they exhibit cell-regulating properties, such as affecting proliferation and osteogenic differentiation of human bone marrow stromal cells and osteoclastogenesis [8,9,10,11,12]. For the success of a bone implant, macroporosity and pore sizes in bone-relevant ranges are critical parameters that were, so far, lacking in the case of monolithic silica/collagen Xerogels. Macroporosity, interconnectivity of pores, and pore sizes can be controlled easily and precisely by additive manufacturing. Moreover, the fabrication of implants with patient-specific shapes of fractures or lesions is possible by using extrusion-based 3D printing. Thus, the advantage of excellent material properties of multiphasic silica/collagen Xerogels was to be combined with the advantages of 3D printing.

Out of the three main techniques of 3D bioprinting—material jetting, material extrusion, and vat polymerization—extrusion-based 3D printing, also 3D plotting, was chosen because of the planned composition of the scaffold. The aim was to have the silica/collagen Xerogel as the effective main component that gives the cell-regulating properties to the later macroporous scaffold. Material jetting, in combination with silica/collagen Xerogels, was not suitable. Xerogels should have a representative size, to give, especially, their cell-regulating properties to the scaffold. Vat polymerization could be suitable, using a photo-curable polymer matrix. Until now, vat polymerization for bone tissue engineering is limited in terms of the amounts of additives, e.g., ceramic powder [20]. By using extrusion-based 3D-printing, Xerogel granules can be used as the filler material incorporated in a matrix suitable for plotting.

Granular silica/collagen Xerogels were incorporated in a high amount, as an effective main component embedded in a viscoelastic matrix. Among the high amount of granules, irregular and ridged shapes of these granules (Figure 4 and Figure 5) are a challenge for the extrusion based plotting process. Owing to its viscoelastic properties, alginate shows good handling for 3D plotting. By mixing Xerogel granules as disperse phase with an alginate matrix as continuous phase in various mass ratios, as well as with different concentrations of the alginate, Xerogel/Alg pastes (biomaterial inks) were prepared. The present study demonstrates that these Xerogel/Alg pastes were suitable for extrusion through conical needles, using a 3D plotter.

The main task for implementation of the 3D-plotting process was the optimization of the mechanical properties of the biomaterial to be plotted. Therefore, the biomaterial ink has to fulfill two requirements. First, the biomaterial ink should exhibit shear-thinning flow behavior to enable 3D plotting under low pressure. Second, biomaterial ink should be elastic enough for the fabrication of stable scaffold strands, as well as for dimensional stability of scaffold geometry after extrusion [23,24]. Shear-thinning behavior was observed for all pastes based on both 13 and 17 wt.% alginate (Figure 2a,b). Hence, all tested Xerogel/Alg pastes are suitable for the processing under low pressure. The evidence of this was provided with the execution of the process using a 3D plotter.

In order to evaluate viscoelasticity of the pastes and to predict dimensional stability of the extruded scaffold strands after 3D plotting, storage modulus G’, and loss modulus G’’ of the shear modulus were measured. The storage modulus G’ indicates elastic deformability, and the loss modulus G’’ indicates viscous deformability to the viscoelasticity of the paste. Thus, a comparison of both shear moduli at low shear stress provides information on the dimensional stability. For G’ > G’’, elastic contribution of viscoelasticity dominated and extruded strands will therefore be dimensionally stable. That was predicted by amplitude sweep tests (Figure 2c,d) for all Xerogel/Alg pastes made of 17 wt.% alginate (component ratios 1:3.33 to 1:2), as well as for Xerogel/Alg pastes made of 13 wt.% alginate, with component ratios of 1:2.5 and 1:2. SEM images of scaffolds fabricated with Xerogel/Alg pastes of 17 wt.% alginate show that strands collapsed after extrusion (Figure 4). Most probably, this is a result of gravity. In contrast to observations in the present study, it has been reported that pure alginate at concentrations of 16.7 wt.% in PBS was processed successfully by 3D plotting [22].

For the latter, 13 wt.% alginate with component ratios of 1:2.5 and 1:2, this could be proved by generated 3D scaffolds with excellent dimensional stability (Figure 3 and Figure 5). In the opposite case, when G’’ > G’, viscous contribution of viscoelasticity dominates and extruded strands will not be stable as present for pure 13 wt.% alginate and pure 17 wt.% alginate. Beside macroscopic observation, SEM images (Figure 5) confirm the dimensional stability of Xerogel/Alg scaffolds.

In general, viscous contribution of viscoelasticity dominates for pastes of pure alginate (13 and 17 wt.%). When comparing pastes of different alginate concentrations, it has to be mentioned that the increase in alginate concentration from 13 to 17 wt.% led to an increase in shear modulus at all, as well as to approximately similar values of G’ and G’’ in the idle state. It can be assumed that the increased density of polymer chains led to a higher degree of formed inter- and intramolecular hydrogen bonds between homopolymeric chain segments. Moreover, rotation of blocks of α-L-guluronic acid is restricted. Both may cause a higher chain immobility and chain stiffness at the molecular level, which macroscopically leads to an increase in both modules with a higher increase in the storage module G’ in the linear viscoelastic region [25].

For all Xerogel-containing pastes, except 13 wt.% alginate with component ratio of 1:3.33, G’ was higher than G’’ (G’ > G’’). Hence, the elastic contribution of viscoelasticity dominated in the Xerogel-containing pastes. Possibly, the irregular and ridged shape of the Xerogel granules causes physical interactions between the Xerogel and the alginate matrix contributing to the dimensional stability after plotting. Strongest characteristic of viscoelastic behavior was observed for 17 wt.% alginate with component ratio of 1:2. For that Xerogel/Alg paste, G’ was much higher than G’’, leading to higher shear stress that was necessary to extrude this paste through conical dispensing needles of size G22 (inner diameter of 410 µm).

After that successful proof of plottability further, investigation on biocompatibility, degradation and mechanical properties will be performed in the future. The properties of the Xerogel [8,9,10,12] as the main component will mainly influence biocompatibility, degradation and mechanical properties of the 3D-plotted Xerogel/Alg scaffolds. Moreover, the plotting process will be investigated in-depth by optical coherence tomography.

The present study is a proof of principle that demonstrates the suitable processing of silica/collagen Xerogel granules via 3D plotting. These composite granules are hybrid materials consisting of several microstructural levels [8]. As shown by other studies, 3D plotting of less complex materials, such as hydroxyapatite or bioglass particles [26,27,28], can be used to generate additional macrostructural levels. In the present case, 3D-plotting of silica/collagen hybrids generates two further levels: (1) hybrids embedded in the alginate matrix (called double-hybrid) and (2) double-hybrids processed to 3D scaffolds consisting of a tailorable solid phase and a continuous pore phase.

## 5. Conclusions

Silica/collagen Xerogel granules were successfully processed into scaffolds for bone substitution by 3D plotting within this study. Alginate was used as continuous phase of the paste, in which silica/collagen Xerogel granules were embedded, in high amounts, as the disperse phase. The resulting Xerogel/Alg pastes were shown to be suitable for 3D plotting, especially based on the viscoelastic properties of Alg. Xerogel/Alg pastes containing a matrix of 13 wt.% alginate in PBS and Xerogel granules with a component ratio of 1:2 were identified to be best suitable for 3D plotting. Scaffolds made of this paste showed lateral macropores resulting from regularly extruded strands. Scaffolds made of these pastes contain Xerogel granules even present at the surface as active major component in a continuous alginate matrix that binds the granules in the scaffold.

## Figures and Tables

**Table 1:** Summary of plotting parameters.

Alg Concentration/wt.%	Xerogel/Alg Ratio	Needle Size (Diameter)/µm	Air Pressure/kPa	Plotting Speed/mm s^−1^
13	1:2	580	500	2
13	1:2.5	410	280	2
13	1:3.33	410	150	3
13	0:1	410	70	4
17	1:2	840	510	0.5
17	1:2.5	410	540	1.5
17	1:3.33	410	350	3
17	0:1	410	130	4

**Table 2:** Measured strand diameter (mean and standard deviation) in the as-printed state, as well as in the dry state. In case, no single strands were present after printing, the strand diameter is not available (n.a.).

Alg Concentration/wt.%	Xerogel/ Alg Ratio	Needle Size (Diameter)/µm	Strand Diameter (as Printed)/µm		Strand Diameter (after Drying)/µm	
			Mean	SD	Mean	SD
13	1:2	580	579	54	537	8
13	1:2.5	410	529	47	351	7
13	1:3.33	410	632	82	409	26
13	0:1	410	n.a.	-	n.a.	-
17	1:2	840	1058	52	712	53
17	1:2.5	410	635	88	508	13
17	1:3.33	410	607	41	470	25
17	0:1	410	n.a.	-	n.a.	-

## Data Availability

The data presented in this study are available on request from the corresponding author.
